# Preparation of Phenolic Aerogel/Quartz Fiber Composites Modified with POSS: Low Density, High Strength and Thermal Insulation

**DOI:** 10.3390/polym18030387

**Published:** 2026-01-31

**Authors:** Xiang Zhao, Dayong Li, Meng Shao, Guang Yu, Wenjie Yuan, Junling Liu, Xin Ren, Jianshun Feng, Qiubing Yu, Zhenyu Liu, Guoqiang Kong, Xiuchen Fan

**Affiliations:** 1 Shandong Institute of Nonmetallic Materials, Jinan 250031, China; zhaoxiang9495@163.com (X.Z.);; 2 Key Laboratory for Liquid Solid Structural Evolution and Processing of Materials, School of Materials Science and Engineering, Shandong University, Jinan 250061, China; 3 Beijing Xinfeng Aerospace Equipment Co., Ltd., Beijing 100089, China

**Keywords:** polyhedral oligomeric silsesquioxane (POSS), phenolic aerogel, click chemistry, thermal insulation, low density

## Abstract

To meet the requirements of next-generation spacecraft thermal protection systems for lightweight materials with high strength, effective thermal insulation, and superior ablation resistance, a novel POSS-modified phenolic aerogel/quartz fiber composite (POSS-PR/QF) was developed using a thiol–ene click reaction combined with a sol–gel process. Covalent incorporation of polyhedral oligomeric silsesquioxanes (POSS) into the phenolic matrix effectively eliminates nanoparticle aggregation and improves interfacial compatibility. As a result, the modified resin is suitable for resin transfer molding (RTM) processes. The resulting composite exhibited an aerogel-like porous structure with enhanced crosslinking density, thermal stability, and oxidation resistance. At 7.5 wt% POSS loading, the composite achieved low density (~0.7 g·cm^−3^) and outstanding mechanical properties, with tensile, flexural, compressive, and interlaminar shear strengths increased by 114%, 79%, 29%, and 104%, respectively. Its thermal conductivity (0.0619 W/(m·K)) and ablation rates were also markedly reduced. Mechanistic studies revealed that POSS undergoes in situ ceramification to form SiO_2_ and SiC phases, which create a dense protective barrier. In addition, this ceramification process promotes char graphitization, thereby enhancing oxidation resistance and thermal insulation. This work provides a promising approach for designing lightweight, high-performance, and multifunctional thermal protection materials for aerospace applications.

## 1. Introduction

Ablative materials are indispensable components of thermal protection systems (TPS) for spacecraft operating in extreme environments, such as atmospheric re-entry and hypersonic flight. Typical examples include fiber-reinforced phenolic composites, carbon–carbon (C/C) composites, and ceramic matrix composites [1,2,3]. Among them, fiber-reinforced phenolic composites are widely used due to their outstanding thermal stability, ablation resistance, and char-forming ability. Nevertheless, with the rapid increase in flight velocity, the requirements for structural lightweighting, superior thermal insulation efficiency, and high-temperature oxidation resistance have intensified [4,5]. Conventional fiber-reinforced phenolic composites are increasingly inadequate. This limitation arises from the intrinsic brittleness, relatively poor mechanical properties, high density, and limited oxidation resistance of phenolic resins, which restrict their applicability in next-generation thermal protection systems [6,7].

To address these challenges, the introduction of nanoscale fillers into phenolic resins has emerged as a promising strategy. Numerous studies have demonstrated that metallic and inorganic nanoparticles, such as SiC, ZrB_2_, Al_2_O_3_, and carbon nanotubes [8,9,10], can markedly enhance the thermal stability, oxidation resistance, and mechanical performance of phenolic-based composites. For example, Paglia et al. [11] reported that the addition of nano-Al_2_O_3_ reduced the linear ablation recession of carbon/phenolic composites from 3.4 mm to 2.0 mm. Pulci et al. [12] achieved up to 115% and 110% improvements in flexural and compressive strength, respectively, by incorporating surface-functionalized nano-ZrO_2_ into low-density (~0.3 g/cm^3^) carbon/phenolic ablators, emphasizing the reinforcement effect of nano-ZrO_2_. Mechanistic studies further revealed that nanoscale additives can densify the char layer, act as effective thermal barriers, and facilitate the in situ formation of protective ceramic layers. In addition, certain nanoparticles regulate the crosslinking and microstructural evolution during carbonization, thereby improving the structural integrity and ordering of residual char, which in turn enhances corrosion resistance and thermal insulation [13,14]. For instance, Wu et al. [15] demonstrated that catalytic pyrolysis induced by copper salts promoted graphitization, reduced pore volume, and significantly improved the oxidation resistance of phenolic-derived carbon. Despite these advances, achieving a balance between property enhancement, density reduction, and resin compatibility remains a formidable challenge, as issues such as filler agglomeration and embrittlement persist [16,17].

Polyhedral oligomeric silsesquioxane (POSS), an organic–inorganic hybrid nanoparticle featuring a rigid Si–O–Si cage core with tunable peripheral organic groups, has recently gained increasing attention as a versatile modifier [18]. POSS combines the thermal and chemical stability of inorganic silica with the compatibility and tailorability of organic moieties, offering exceptional structural design flexibility. In phenolic systems, POSS has been shown to enhance char layer integrity, suppress thermal conductivity, and improve mechanical strength through molecular-level dispersion and strong interfacial interactions [19]. For instance, Wu et al. [20] demonstrated that octaacetylstyrene-functionalized POSS (AS-POSS) formed hydrogen bonds with phenolic resins, generating a stable nanoscale miscible system at low POSS contents as confirmed by FTIR analysis. These findings suggest that POSS is a particularly promising candidate for reinforcing phenolic resins under harsh thermo-oxidative environments.

In this study, we report the design and fabrication of a novel POSS-modified porous phenolic/quartz fiber (POSS–PR/QF) composite that simultaneously achieves low density, high mechanical strength, and outstanding oxidation and ablation resistance. POSS molecules were covalently incorporated into the phenolic matrix via thiol–ene click chemistry, effectively mitigating the incompatibility and aggregation issues commonly encountered with inorganic nanoparticles. Furthermore, a facile sol–gel and ambient-pressure drying process was adopted to introduce an aerogel-like porous architecture, which significantly reduced composite density while maintaining structural robustness. The resulting composites exhibited superior mechanical and thermal performance, as well as remarkable oxidation and ablation resistance. These results provide new insights and practical strategies for the development of next-generation lightweight and high-efficiency thermal protection materials.

## 2. Materials and Methods

### 2.1. Experimental Materials and Characterization Methods

(3-Mercaptopropyl)trimethoxysilane and o-allylphenol were purchased from MacLean Company (Shanghai, China) and used as received. Quartz fiber needle punched preform (160 × 160 × 10 mm) was purchased from Feilihua Quartz Glass Co., Ltd. (Jingzhou, China) with an apparent density of 0.35 g/cm^3^. Hexamethylenetetramine (HMTA) was provided by East China University of Science and Technology, Shanghai. 37% hydrochloric acid, methanol, dichloromethane, tetrahydrofuran, anhydrous magnesium sulfate, phenol, 37% formaldehyde aqueous solution, and dimethyl benzoate (DMPA) were purchased from commercial suppliers and used without further purification.

TGA: Thermal stabilities of resins were performed by thermogravimetric analysis (TGA) in a NETZSCH STA 449c/3/G thermal system under air or N_2_ atmosphere. The analysis was performed at a constant heating rate of 10 °C/min with flow rate of about 60 mL/min with temperature ranges from 35 °C to 1000 °C.

Ablation test: Ablation abilities of composites were carried out under an oxyacetylene torch, and a test sample of Φ30 × 10 mm was subjected to the torch for 30 s, according to GJB 323A-96 [21]. The heat flux was 4.2 ± 0.2 MW/m^2^. The flow rates of oxygen and acetylene were 1512 L/h and 1116 L/h. The gas pressures of oxygen and acetylene were 0.40 MPa and 0.095 MPa, respectively. The oxyacetylene gun with 2 mm diameter was vertical to the surface of sample, and the distance from gun to the surface of sample was 10 mm. The ablative property of the sample is characterized by linear ablation rate (LAR) and mass ablation rate (MAR). LAR is the change in material thickness before and after ablation measured by micrometer with an accuracy of 0.001 mm, while MAR is the change in mass measured by analytical balance with an accuracy of 0.001 g, as shown in Equations (1) and (2), where *dl* and *dm* represented the decline in length and weight of the samples with respect to the changes in testing time *dt*, respectively.

(1) LAR=dl/dt

(2) MAR=dm/dt

DSC: Differential scanning calorimetry (DSC) was performed on a Mettler Toledo DSC3 over the range from 25–300 °C at a heating rate of 5, 10, 15, and 20 °C/min in N_2_.

Thermal conductivity: Thermal conductivity of the samples were acquired through thermal constant analyze equipment (TPS 2500, Hot Disk AB, Gothenburg, Sweden) according to GB/T 10294-2008 [22], with the samples’ dimension of 160 mm × 160 mm × 10 mm under the standard temperature and pressure.

FT-IR: Fourier transform infrared spectroscopy (FT-IR) measurements were performed on Tensor 27 spectrometer (Bruker Corporation, Billerica, MA, USA) by using KBr disks at the ambient temperature with the wavelength at the range of 400–4000 cm^−1^.

XPS: X-ray photoelectron spectroscopy (XPS) was recorded on the AXIS ULTRA DLD instrument (Kratos Analytical Ltd., Manchester, UK) with Al Kα ray. All spectra were charge referenced against the C1s peak at 284.8 eV to correct for charging effects during acquisition.

NMR: The ^1^H (300 MHz), ^13^C (101 MHz), and ^29^Si NMR (79 MHz) spectra were recorded with a Varian XL 300 MHz spectrometer and Varian VNMR-S 400 MHz spectrometer with samples in CDCl_3_ solution. The chemical shifts are reported in ppm and were referenced to the residual solvent signals (δH = 7.26 ppm, δC = 77.36 ppm for CDCl_3_).

SEM: Surface morphology was observed on FEI Instrument Verios G4 ultra-high resolution field emission scanning electron microscope (SEM, Thermo Fisher Scientific, Hillsboro, OR, USA) at an acceleration voltage of 25 kV. Energy dispersive spectroscopy (EDS) was performed on an Oxford INCA X-ACT (Oxford Instruments, Abingdon, UK) attaching to the SEM apparatus.

XRD: X-ray diffraction (XRD) measurements were carried out by a Bruker D8 Advance spectrometer (Bruker AXS GmbH, Karlsruhe, Germany) with Cu Kα radiation (0.154 nm, 40 kV, 40 mA). Every sample was scanned from a 2θ angle of 10–90° at a scan rate of 5°/min.

Raman spectroscopy spectra: Raman spectroscopy spectra was recorded from 500 to 2000 cm^−1^ using a Raman spectrometer (Alpha300R, WITec GmbH, Ulm, Germany) with λ = 514.5 nm.

Mechanical properties: Flexural strength, interlaminar shear strength (ILSS), tensile strength and compression strength of the samples were measured using electron omnipotence experiment machine SANS-CMT8505 (Shenzhen New Sansi Co., Shenzhen, China) according to GB/T 1449-2005 [23], GB/T 1450.1-2005 [24], GB/T 1447-2005 [25] and GB/T 1448-2005 [26], respectively.

GPC: Gel permeation chromatography (GPC) analysis was performed using a Waters 1515 Instrument (Waters Corporation, Milford, MA, USA) using tetrahydrofuran as the eluent and polystyrene as the standard for calibration.

BET: The specific surface area and pore structure of the samples were characterized by nitrogen adsorption–desorption measurements using a surface area and porosity analyzer (BET). Prior to the measurements, all samples were degassed under vacuum at 120 °C for at least 8 h to remove physically adsorbed moisture and residual solvents. Nitrogen adsorption–desorption isotherms were recorded at 77 K. The specific surface area was calculated using the Brunauer–Emmett–Teller (BET) method in the relative pressure (P/P_0_) range of 0.05–0.30. The total pore volume was determined from the amount of nitrogen adsorbed at a relative pressure close to 0.99. The pore size distribution was derived from the adsorption branch of the isotherm using the Barrett–Joyner–Halenda (BJH) model.

### 2.2. Synthesis of Mercapto Polyhedral Oligomeric Silsesquioxne (SH-POSS)

**Figure 1:**
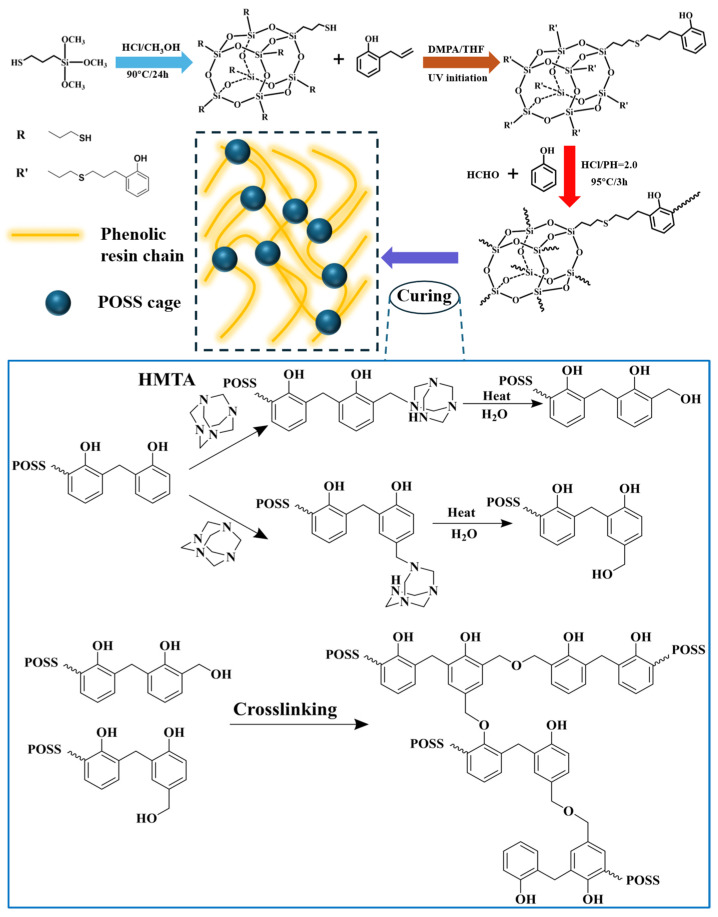
Synthesis of SH-POSS and Chemical Modification of Phenolic Resin.

According to literature reports [27,28], octamercapto oligomeric silsesquioxane (SH-POSS) was prepared by hydrolysis and condensation reaction of (3-mercaptopropyl)trimethoxysilane catalyzed by concentrated hydrochloric acid. The synthesis route is shown in Figure 1. Specifically, 360 mL of methanol, 15 mL of (3-mercaptopropyl)trimethoxysilane, and 30 mL of 37% hydrochloric acid were added to a 1000 mL three-necked flask. After stirring and mixing, the temperature was raised to 90 °C and refluxed for 24 h. After the reaction was completed, the mixture was refrigerated and allowed to stand for 1 h. A milky white precipitate was precipitated at the bottom of the flask. The supernatant was poured out, a small amount of dichloromethane was added to fully dissolve the precipitate, and then an appropriate amount of icy methanol was added to precipitate the product. The above process was repeated three times to obtain a crude product. The crude product was dissolved in dichloromethane and washed with deionized water. The dichloromethane phase was then separated and dried over anhydrous magnesium sulfate to remove residual water. After filtration, the solvent was evaporated to dryness to obtain SH-POSS with a yield of 74.5%.

### 2.3. Synthesis of POSS-Modified Phenolic Resin

**Figure 2:**
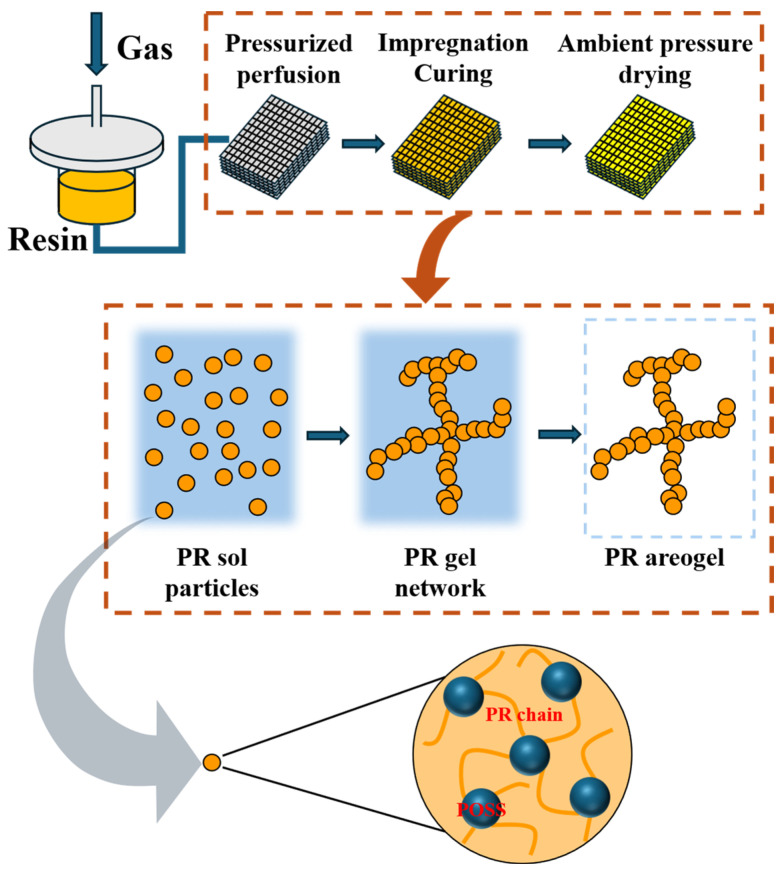
Preparation process of phenolic aerogel composite materials.

With SH-POSS and o-allylphenol as reactants, DMPA as photoinitiator, and dichloromethane as solvent, phenolic polyhedral oligomeric silsesquioxane (Phenolic-POSS) was synthesized based on the click reaction between thiol groups and electron-rich double bonds [29,30] and POSS-modified phenolic resin was further synthesized. The synthesis route is shown in Figure 2. Specifically, 10 g of SH-POSS and 11 g of o-allylphenol were dissolved in 200 mL of THF. Oxygen was removed by purging with nitrogen for 30 min, and subsequently, 1 g of DMPA was added under a nitrogen atmosphere. The mixture was reacted under 365 nm ultraviolet light for 2 h. The product was washed with deionized water, and the organic phase was dried to obtain a yellow viscous liquid product Phenolic-POSS (20.1 g, 95.7%). 94.1 g (1 mol) of phenol was heated and dissolved in a 500 mL three-necked flask. Phenolic-POSS with a phenol mass fraction of 2.5% to 10% (2.5–10%POSS-PR) was dissolved in the hot phenol. 68.9 g of 37% formaldehyde solution and an appropriate amount of hydrochloric acid were added, and the pH was adjusted to 1.5–2.0. The mixture was mixed and stirred until uniform, with the molar ratio of phenol to formaldehyde being 1:0.85. The mixture was heated to 90 °C and refluxed for 3 h. Water was then removed by vacuum distillation. After completion, the hot reaction product was poured from the flask, cooled, and sealed for later use (approximately 150 g of resin product). A pure phenolic blank control was prepared. The synthesis steps remained unchanged except for the addition of Phenolic-POSS.

### 2.4. Preparation of Phenolic Aerogel/Quartz Fiber Composites

The fabrication process of the aerogel composite is illustrated in Figure 2. An ethanol solution of phenolic resin with a solid content of 40% was prepared, and hexamethylenetetramine (HMTA, 10 wt% relative to the mass of phenolic resin) was introduced as the curing agent. Phenolic aerogel/quartz fiber composites (PR/QF) were then produced through a sol–gel process followed by ambient pressure drying. The fiber preform was placed into a customized mold, and resin transfer molding was employed to achieve complete infiltration of the resin into the fiber preform. Specifically, compressed air is used to pressurize the resin tank to 1.5–2 atm. Under pressure, the resin solution is injected into a sealed mold with an inlet/outlet. The mold contains a quartz fiber preform. The injection is slow and uniform to ensure that the resin fully impregnates the fiber preform and to expel any residual air from the mold. After about 1 h of injection and impregnation, the mold inlet/outlet is closed and the sealed mold was transferred to an oven for sol–gel transition and curing reactions (the detailed curing process is shown in Figure S1 of the Supplementary Material). After curing, the composite was dried at 60 °C for 24 h to obtain the phenolic aerogel/quartz fiber composite.

## 3. Results and Discussion

### 3.1. Characterization of SH-POSS, Phenolic-POSS and POSS-PR

**Figure 3:**
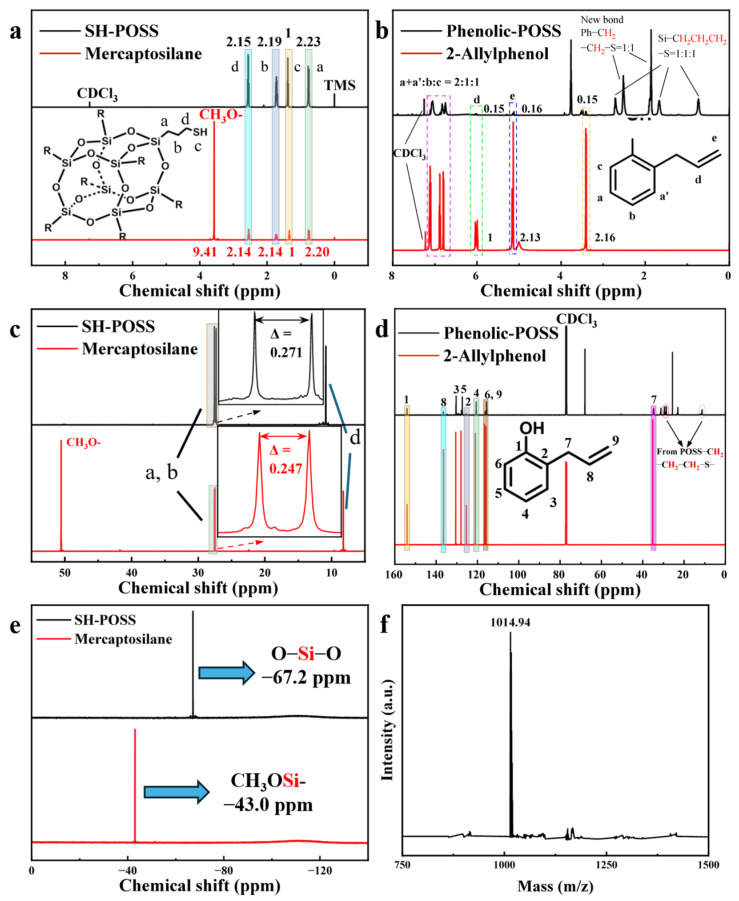
NMR spectra of SH-POSS and Phenolic-POSS: (**a**,**b**) ^1^H NMR; (**c**,**d**) ^13^C NMR; (**e**) ^29^Si NMR; (**f**) ESI-MS of SH-POSS.

The molecular structures of SH-POSS and Phenolic-POSS were characterized by ^1^H NMR, ^13^C NMR, and ^29^Si NMR, as shown in Figure 3. In Figure 3a, comparison between SH-POSS and mercaptosilane spectra reveals that the signals at 0.75 ppm (a), 1.72 ppm (b), 1.38 ppm (c), and 2.57 ppm (d) [31] can be assigned to the four types of protons on the –CH_2_CH_2_CH_2_–SH side chain. The integral area ratio of these peaks (2.15:2.19:1:2.23) is consistent with the theoretical hydrogen ratio (2:2:1:2), confirming the intact preservation of the thiol groups. Meanwhile, the disappearance of the CH_3_O– signal at 3.53 ppm [32] after reaction indicates that the hydrolysis of mercaptosilane proceeded completely to yield SH-POSS. As shown in Figure 3b, comparison of Phenolic-POSS with 2-allylphenol spectra demonstrates that the –SH signal at 1.38 ppm, together with the olefinic signals at 6.02 and 5.15 ppm, nearly vanished after reaction. This disappearance confirms that the thiol and double bond groups underwent a click reaction, resulting in the successful synthesis of Phenolic-POSS. The ^13^C NMR spectra (Figure 3c,d) [31] further corroborate the formation of SH-POSS and Phenolic-POSS. To further verify the Si–O–Si cage framework of POSS, ^29^Si NMR analysis was performed (Figure 3e). The Si resonance of methoxysilane shifted from −43.0 ppm to −67.2 ppm after reaction, which is consistent with literature reports [31] for T8-type POSS, thereby confirming the formation of a T8 cage structure. To verify the purity of the synthesized SH-POSS, ESI-MS analysis was performed (Figure 3f). In the negative ion mode, the dominant molecular ion peak was observed at m/z = 1014.94, while the theoretical molecular weight of SH-POSS is 1015.97. The difference corresponds precisely to the mass of H^+^, consistent with the deprotonation feature of the negative ion mode ([M–H]^−^). This confirms that the synthesized product is predominantly the desired SH-POSS.

**Figure 4:**
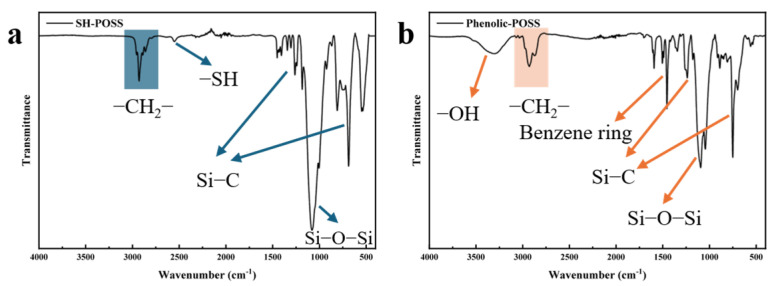
FT-IR spectroscopy: (**a**) SH-POSS; (**b**) Phenolic-POSS.

The molecular structures of SH-POSS and Phenolic-POSS were further investigated using Fourier Transform Infrared (FT-IR) spectroscopy, as shown in Figure 4. In the FT-IR spectrum of SH-POSS, the absorption bands observed in the range of 3000–2800 cm^−1^ are attributed to the stretching vibrations of methylene (–CH_2_–) groups, confirming the presence of alkyl side chains. The characteristic peak appearing at 2551 cm^−1^ corresponds to the stretching vibration of thiol (–SH) groups, indicating that the reactive thiol functionalities were successfully introduced onto the POSS cage. Additionally, the absorption peaks at 1259 and 686 cm^−1^ are assigned to the asymmetric and symmetric stretching vibrations of Si–C bonds, respectively, which verify the covalent linkage between the inorganic silsesquioxane core and the organic substituents. A distinct and intense peak at 1080 cm^−1^ corresponds to the asymmetric stretching vibration of the Si–O–Si framework, a typical feature of the silsesquioxane cage structure, demonstrating the integrity of the inorganic POSS core [33]. In contrast, the FT-IR spectrum of Phenolic-POSS shows several notable differences compared with that of SH-POSS. A broad and intense absorption band appears in the region of 3600–3100 cm^−1^ [34], which can be attributed to the stretching vibration of phenolic hydroxyl (–OH) groups. Moreover, a strong absorption peak emerges at 1457 cm^−1^, corresponding to the characteristic skeletal vibration of the benzene ring, confirming the successful incorporation of phenyl groups. Meanwhile, the disappearance of the –SH stretching vibration peak at 2551 cm^−1^ indicates that the thiol groups have completely participated in the substitution reaction to form Phenolic-POSS. The FT-IR results provide evidences for the successful synthesis of both SH-POSS and Phenolic-POSS, as well as the preservation of the POSS cage framework throughout the chemical modification process. These findings validate the structural integrity and effective functionalization of the POSS derivatives, laying a foundation for their subsequent application in polymer modification and hybrid material design.

**Figure 5:**
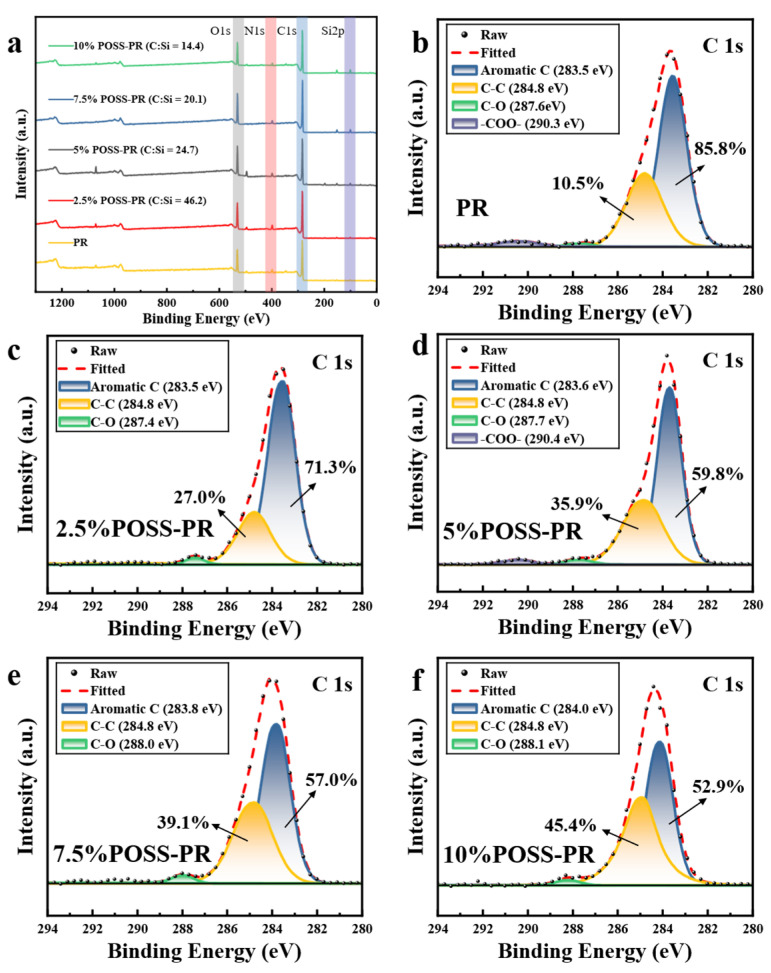
XPS survey spectrum and C 1s XPS spectra of POSS-PR: (**a**) XPS survey spectrum; (**b**) C 1s XPS of PR; (**c**–**f**) C 1s XPS of POSS-PR.

**Figure 6:**
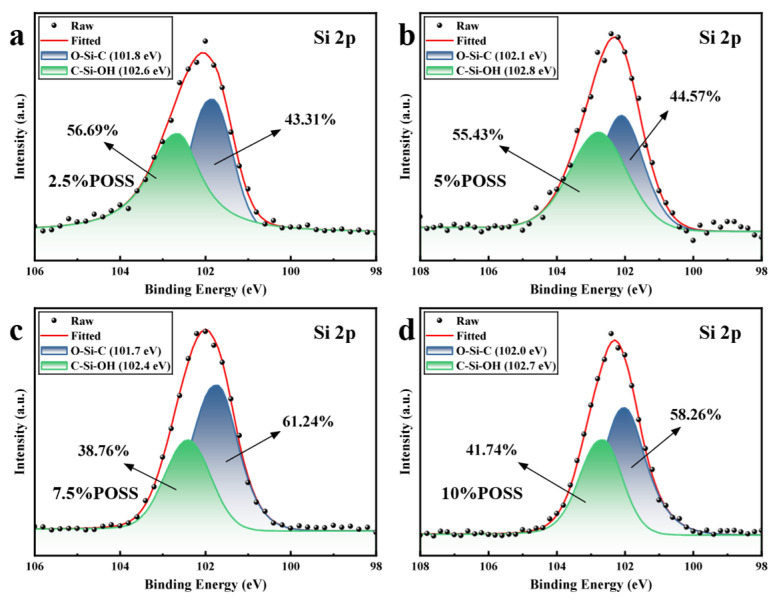
Si 2p XPS spectra of POSS-PR: (**a**–**d**) 2.5–10% POSS-PR.

The cured phenolic resin (PR) and POSS-modified PR with varying POSS loadings were analyzed using X-ray photoelectron spectroscopy (XPS) (Figure 5 and Figure 6). The wide-scan XPS spectra (Figure 5a) reveal a gradual decrease in the C/Si atomic ratio with increasing POSS content, indicating not only the successful incorporation of POSS into the resin matrix but also the effective introduction of silicon species at the molecular level. This suggests that the POSS molecules are uniformly dispersed within the polymer network rather than forming isolated aggregates, which is critical for achieving homogeneous hybrid structures. The C/Si ratio has been shown to play a pivotal role in determining the thermal and oxidative behavior of phenolic resins [33]. Higher silicon content, as introduced via POSS, promotes the formation of a dense, continuous silica (SiO_2_) layer during pyrolysis and high-temperature oxidation. This in situ protective layer acts as a physical barrier, reducing oxygen diffusion and suppressing further decomposition of the carbonaceous matrix, thereby enhancing thermal stability and oxidative resistance [35]. Moreover, tuning the C/Si ratio can influence the microstructure of the resulting char [36], including the development of a more crosslinked Si–O–C network and improved interfacial bonding within the carbonized layer [37]. Such structural reinforcement translates into superior mechanical performance, lower mass loss during thermal degradation, and enhanced ablation resistance under extreme conditions.

The C 1s XPS spectra are shown in Figure 5b–f. The carbon species are mainly present as aromatic C (283.5–284.0 eV) [38], C–C (284.8 eV), C–O (287.4–288.1 eV), and –COO– (290.3–290.4 eV) [39]. With increasing POSS loading, the proportion of aromatic C decreases, while that of aliphatic C increases. This change indicates that the incorporation of Phenolic-POSS introduces new crosslinking sites and connectivity within the resin network, effectively constructing new “bridges” between polymer chains. The synergistic interaction between the POSS cage structure and the aromatic rings of the phenolic resin contributes to the enhancement of mechanical properties and mitigates the intrinsic brittleness of the resin [19].

The Si 2p XPS spectra are shown in Figure 6. Due to the small binding energy difference between the Si 2p1/2 and 2p3/2 orbitals, they are difficult to resolve and thus a single-peak fitting was applied. Silicon is mainly present in the forms of O–Si–C (101.7–102.1 eV) and C–Si–OH (102.4–102.8 eV) [40]. The C–Si–OH species may originate from partially unreacted open-cage POSS in the product. Notably, at a POSS loading of 7.5%, the proportion of O–Si–C reaches its maximum, and the overall oxidation state is relatively low (O–Si–C at 101.7 eV, 61.24%), indicating that the system exhibits enhanced oxidation resistance under these conditions.

### 3.2. Thermal Behavior of POSS-PR

**Figure 7:**
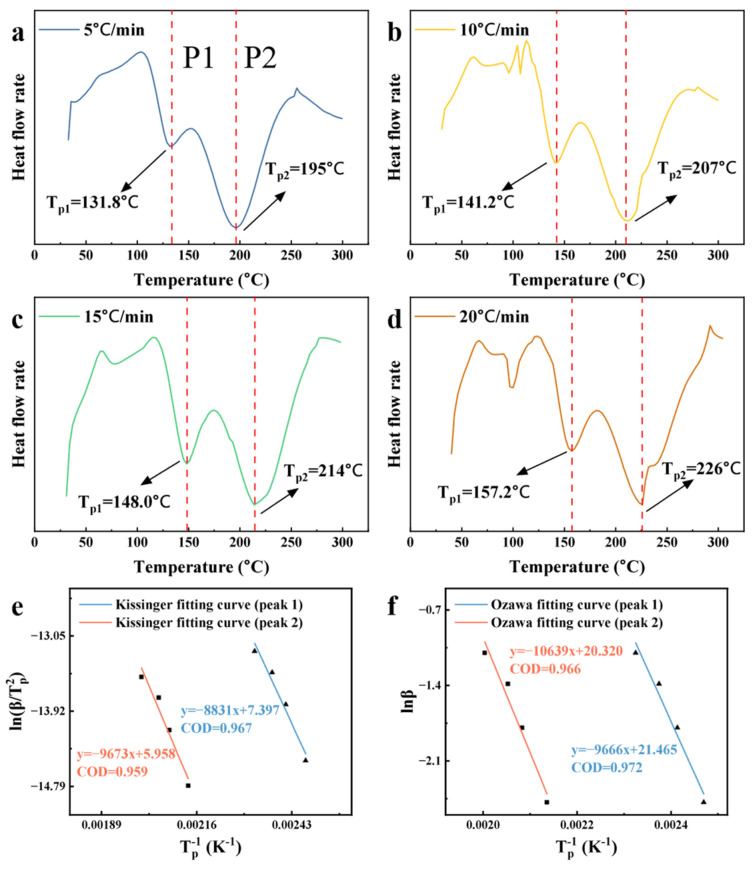
DSC curve of the curing behavior of 7.5% POSS-PR: (**a**–**d**) DSC at different heating rates; (**e**) Kissinger fitting curve; (**f**) Ozawa fitting curve.

To investigate the curing characteristics and optimize the curing process, the phenolic resin containing 7.5 wt% Phenolic-POSS was characterized by differential scanning calorimetry (DSC) (Figure 7). Unlike conventional phenolic resins, the DSC curve of the POSS-modified PR exhibits two distinct exothermic peaks, indicating the presence of two different curing pathways within this hybrid system. The first peak (P1) corresponds to the conventional curing reaction of linear phenolic resin, while the second peak (P2) is attributed to a curing process initiated from the POSS core, in which polymer chains grow radially from the eight corners of the T8 cage structure.

To further elucidate the kinetic behavior of these two curing reactions, P1 and P2 were separately analyzed using the Kissinger, Ozawa, and Crane methods [41], and the corresponding kinetic parameters are presented in Figure 7e,f and Table 1.

The average activation energies (*E*_a_) for both P1 and P2 are lower than those reported for conventional phenolic resin curing reactions in the literature [42], and the apparent reaction orders for both steps are close to one. These observations indicate that the incorporation of POSS facilitates the curing process by reducing the energy barrier, and that the curing reactions of both pathways predominantly follow first-order kinetics.

The observed reduction in activation energy can be rationalized by the unique structural features of the POSS molecule. The rigid T8 cage provides a three-dimensional spatial framework, and the radially oriented R groups offer directional guidance for crosslinking reactions. This geometric arrangement reduces steric hindrance during polymer chain growth. As a result, curing can proceed preferentially via ortho-position reactions, rather than being strictly limited to para-position coupling, as is typical in conventional phenolic resins. This behavior is consistent with literature reports that phenolic resins with a higher degree of ortho-substitution exhibit lower curing activation energies than their standard counterparts [43].

Overall, the DSC and kinetic analysis demonstrate that POSS incorporation not only introduces an additional curing pathway but also enhances the curing efficiency of the phenolic network. The dual-curing mechanism and reduced activation energy collectively contribute to the formation of a highly crosslinked, three-dimensional hybrid network. This network is expected to improve the thermal stability and mechanical integrity of the resulting POSS-PR composites.

**Figure 8:**
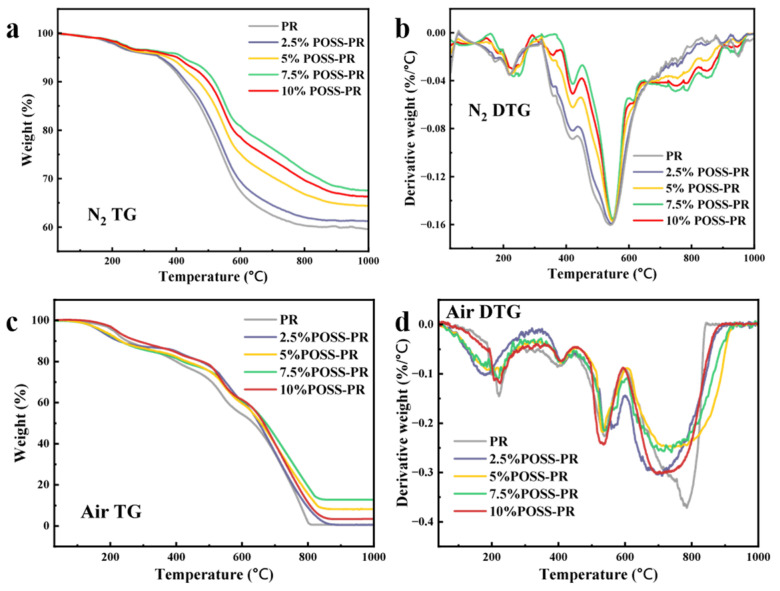
TG and DTG of cured resins under air/nitrogen: (**a**) N_2_ TG; (**b**) N_2_ DTG; (**c**) Air TG; (**d**) Air DTG.

The thermal behavior of the cured resins was investigated by thermogravimetric analysis (TGA) under both nitrogen and air atmospheres (Figure 8 and Table 2). Overall, the POSS-modified phenolic resin (POSS-PR) exhibits significantly enhanced thermal performance compared to the unmodified phenolic resin (PR). Under nitrogen, the initial decomposition temperature (T_5%_) of 7.5 wt% POSS-PR is 421 °C, which is 68 °C higher than that of PR, while the temperature at maximum decomposition rate (T_dmax_) reaches 556 °C, 24 °C higher than PR. The char yield at 800 °C (R_800_) is 58.5%, representing an increase of 7.8% over PR. In an oxidative atmosphere, the R_800_ of POSS-PR is 19.3%, which is 17.4% higher than that of PR. These results collectively indicate a marked improvement in the thermal stability of the resin upon POSS incorporation, particularly under oxidative conditions. This suggests a significant enhancement of thermo-oxidative resistance.

The superior thermal performance of POSS-PR can be attributed primarily to the high bond energy of the Si–O bond (452 kJ/mol [44]), which is substantially higher than that of the C–C bond (346 kJ/mol [45]). Upon thermal oxidation or pyrolysis, POSS undergoes selective oxidative cleavage, generating high-melting-point ceramic species such as SiO_2_ and SiC [46]. These ceramic residues play a dual protective role: first, they consume oxygen in the vicinity of the polymer matrix, and second, they form a dense, continuous oxide layer that acts as a physical barrier [47], inhibiting further thermal and oxidative attack on the underlying resin. Such in situ ceramic formation not only stabilizes the carbonaceous matrix but also contributes to the observed increase in residual mass under both inert and oxidative atmospheres.

### 3.3. Mechanical Properties and Thermal Insulation Properties of POSS-PR Aerogel/QF

**Figure 9:**
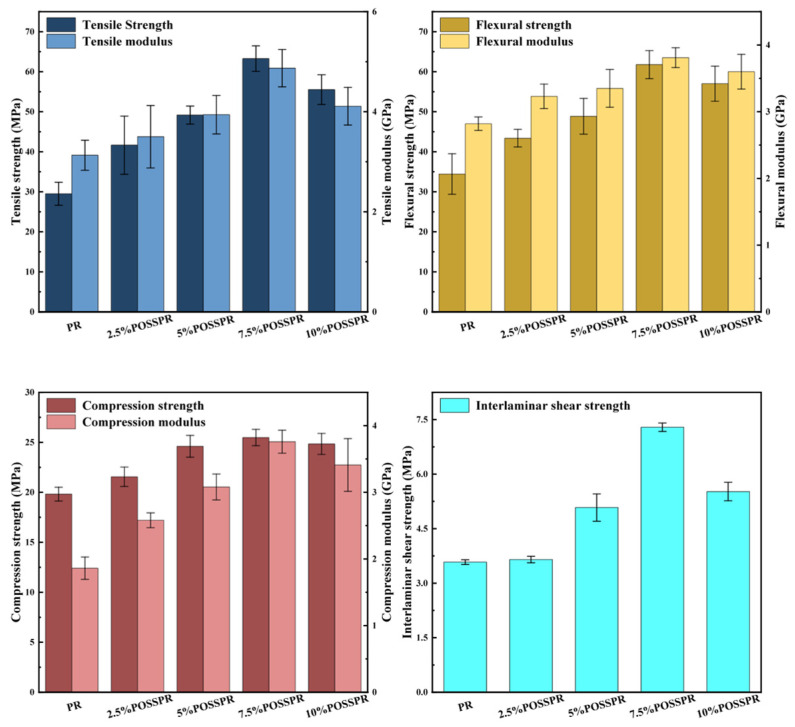
Mechanical performance of fiber-reinforced composites.

The mechanical properties of the prepared POSS-PR aerogel/QF composites, including tensile, flexural, compressive, and interlaminar shear strengths, were systematically evaluated (Figure 9). Among the tested samples, 7.5%POSS-PR/QF exhibited the most outstanding mechanical performance. Specifically, the tensile, flexural, compressive, and interlaminar shear strengths of the 7.5%POSS-PR/QF were 63.26, 61.76, 25.48, and 7.29 MPa, respectively, representing enhancements of 114%, 79%, 29%, and 104% compared with the corresponding values of the unmodified PR/QF (29.5, 34.43, 19.82, and 3.58 MPa). The elastic moduli were also significantly improved, reflecting a more robust and stiff composite network.

Overall, the mechanical strength of the composites exhibited a trend of initially increasing with POSS content, followed by a slight decrease at higher loadings. This behavior can be attributed to the unique T8 cage structure of POSS, which facilitates stress distribution and enhances the crosslinking density within the polymer network, thereby improving load transfer and overall composite integrity [48]. However, excessive POSS loading may induce particle aggregation, leading to local stress concentrations and reduced interfacial adhesion. These effects ultimately diminish the mechanical reinforcement provided by the POSS. These findings indicate that an optimal POSS content exists to maximize the synergistic combination of stress dispersion, crosslinking enhancement, and structural uniformity. This balance enables superior mechanical performance in phenolic resin-based hybrid composites.

**Figure 10:**
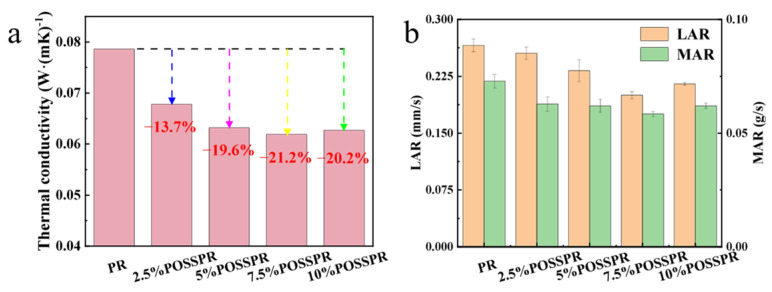
Characterization of thermal insulation and ablation performance: (**a**) thermal conductivity of samples; (**b**) LAR and MAR of samples.

**Figure 11:**
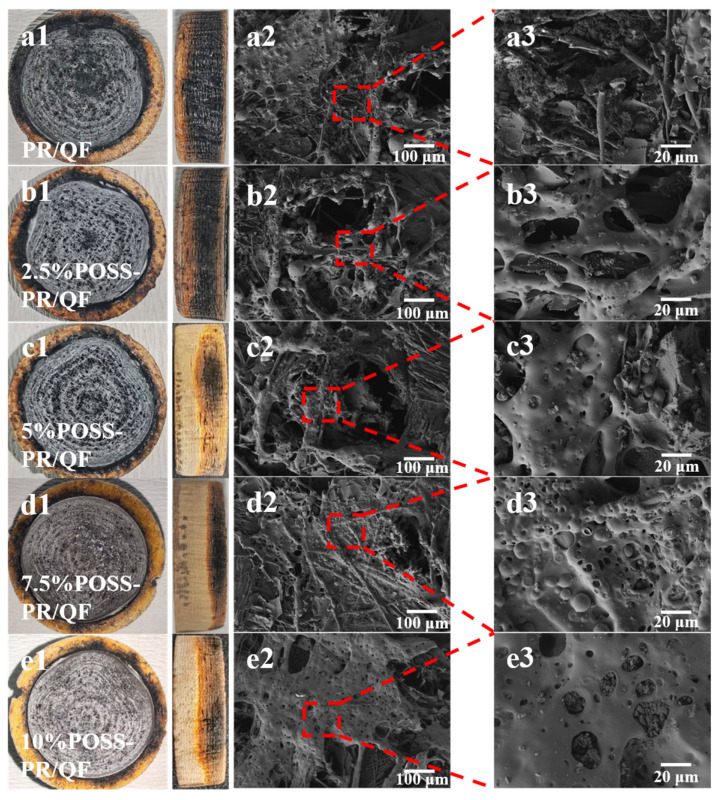
Surface morphology of ablated samples: (**a1**–**e1**) ablation samples of PR/QF and 2.5–10%POSS-PR/QF; (**a2**–**e2**,**a3**–**e3**) microstructure of the ablation samples of PR/QF and 2.5–10%POSS-PR/QF.

To evaluate the thermal insulation and ablation resistance of the composites, thermal conductivity and ablation rates were measured (Figure 10). Both properties promoted with increasing POSS content. For the 7.5%POSS-PR/QF composite, the thermal conductivity decreased to 0.0619 W/(m·K), which is 21.2% lower than that of PR/QF (0.0786 W/(m·K)). Meanwhile, the linear ablation rate (LAR) and mass ablation rate (MAR) decreased to 0.197 mm/s and 0.0584 g/s, corresponding to reductions of 25.9% and 19.8%, respectively, compared with PR/QF (0.266 mm/s and 0.0728 g/s). Typically, thermal insulation and ablation resistance in aerogels are mutually exclusive [49,50], as high porosity and low density favor insulation, whereas ablation resistance generally benefits from higher density. In this study, the POSS-PR/QF system simultaneously enhances both properties. The front and side photographs of the ablation samples are shown in Figure 11(a1–e1). Due to the harsh conditions of the oxyacetylene ablation test (flame temperature up to 3000 °C, heat flux density 4.2 MW), the sample surface suffered severe ablation and cracking, exhibiting a concave shape. This is attributed to the lower ablation resistance and strength of low-density materials compared to high-density materials (such as molded products). Furthermore, the side photographs show that the composite with 7.5 wt% POSS addition exhibits better ablation resistance. The black and white interlocking ring-shaped textures on the surface originate from the phenolic carbonized layer and fused silica fibers. The pure PR and low-POSS composites predominantly undergo surface carbonization, while composites with 7.5 wt% and 10 wt% POSS loadings develop continuous and uniform white fused SiO_2_ ceramic layers. The formation of such protective ceramic layers represents the macroscopic mechanism responsible for the improved ablation performance and oxidation resistance of POSS-modified phenolic resins.

BET analysis (shown in the Supplementary Materials Figures S5–S8) suggests that the material maintains porosity while significantly reducing pore size and increasing specific surface area, which hinders convective heat transfer. Under oxy-acetylene flame temperatures of 2000–3000 °C, POSS undergoes oxidative decomposition to form molten ceramic species such as SiO_2_ and SiC, which deposit on the surface to create a dense protective layer, effectively suppressing internal ablation.

### 3.4. Microstructure of the Composite Material After Ablation

To elucidate the ablation mechanism of the POSS-PR/QF composites, the morphology of the post-ablation surfaces was examined by scanning electron microscopy (SEM) following oxyacetylene testing (Figure 11). The unmodified PR/QF composites exhibited severe surface degradation, characterized by abundant voids and extensive fiber breakage, indicating poor structural integrity under high-temperature ablation conditions. In contrast, the POSS-PR/QF composites displayed significantly more intact ablation surfaces, with fibers well preserved within the matrix. Notably, the 7.5%POSS-PR/QF sample exhibited a uniform surface morphology with clear attachment of ceramic-like particles on the resin surface, demonstrating that POSS undergoes high-temperature transformation into thermally stable species such as SiO_2_ and SiC [33,51]. The formation of this in situ ceramic oxide layer effectively shields the underlying polymer from direct thermal erosion, thereby enhancing both ablation resistance and oxidative stability. These observations are consistent with the measured ablation rate data.

**Figure 12:**
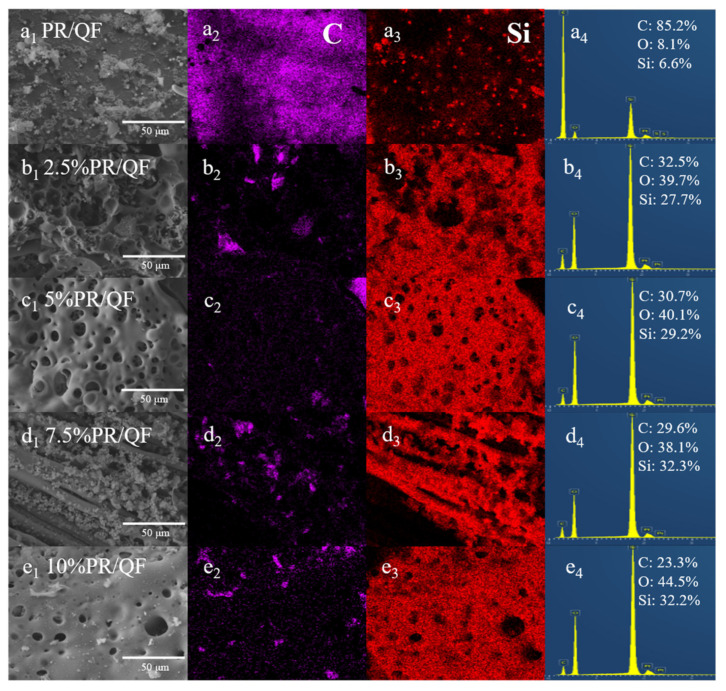
Elemental analysis of ablation sample surface: (**a1**–**e1**) ablation surfaces of PR/QF and 2.5–10%POSS-PR/QF; (**a2**–**e2**) C element EDS analysis of PR/QF and 2.5–10%POSS-PR/QF; (**a3**–**e3**) Si element EDS analysis of PR/QF and 2.5–10%POSS-PR/QF; (**a4**–**e4**) element distribution of PR/QF and 2.5–10%POSS-PR/QF.

Energy-dispersive X-ray spectroscopy (EDS) was further performed to analyze the elemental composition of the ablated surfaces (Figure 12). As the POSS content increased, the carbon content decreased while the silicon content increased, in agreement with the previous XPS results. This compositional trend indicates a transition in the ablation mechanism: from the conventional formation of a carbonaceous char layer in PR/QF composites to the generation of a silicon-rich ceramic protective layer in POSS-PR/QF composites. The presence of such a ceramic layer provides enhanced thermal protection and reinforces the surface against oxidative attack [19,37]. This behavior confirms the critical role of POSS in improving high-temperature performance and prolonging the service life of materials under extreme conditions.

**Figure 13:**
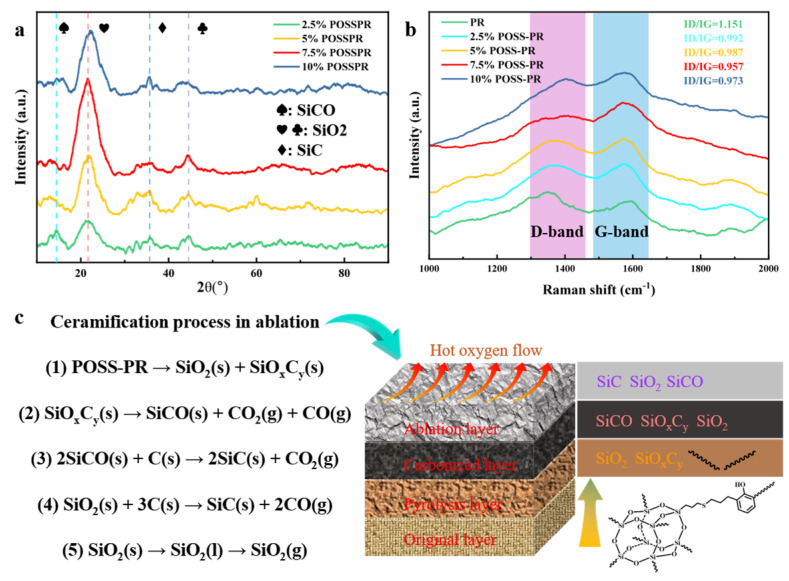
Changes in the molecular structure of the ablated sample: (**a**) XRD analysis; (**b**) Raman analysis; (**c**) ablation mechanism.

To gain deeper insight into the molecular-scale mechanisms governing the ablation behavior of the POSS-PR/QF composites, X-ray diffraction (XRD) and Raman spectroscopy were employed to characterize the post-ablation residues, as illustrated in Figure 13. The XRD patterns demonstrate that during the high-temperature oxidative degradation process, the POSS-PR/QF system predominantly yields crystalline phases of SiO_2_, SiC, and SiCO. With increasing POSS content, the diffraction peak at 2θ = 21.5°, corresponding to the β-quartz phase of SiO_2_, gradually intensifies and becomes most pronounced at a POSS loading of 7.5%. Meanwhile, the peak at 2θ = 35.7°, assigned to the α-SiC phase, also increases in intensity and reaches its maximum at 10% POSS content. In contrast, the diffraction peak located at 2θ = 8.17°, ascribed to SiCO, exhibits a progressive decline.

These observations imply that the thermal degradation of POSS undergoes a multi-step transformation process (Figure 13c): initially forming SiCO as an intermediate phase, subsequently converting into high–melting point SiO_2_, and finally evolving into thermally robust SiC at elevated temperatures. Such a phase transformation pathway aligns well with the ceramization mechanism commonly reported for silicon-containing polymer-derived ceramics [33,37,41,52], where SiCO serves as a transitional phase prior to SiC and SiO_2_ formation under oxidative and high-temperature environments. The generation of SiC and SiO_2_ not only enhances the thermal stability of the residue but also contributes to the structural integrity of the protective char layer, thereby improving the ablation resistance of the composite system.

The Raman spectra further elucidate the carbon structure evolution within the ablated char. In carbon-containing materials, two prominent characteristic bands are typically observed: the D-band around 1300 cm^−1^, associated with disordered carbon structures, and the G-band near 1580 cm^−1^, related to graphitic carbon [53,54]. The intensity ratio ID/IG is widely recognized as a diagnostic parameter for assessing the degree of graphitization, where a lower ratio reflects a higher level of structural ordering and graphitic domain formation. As depicted in Figure 13b, the ID/IG ratio decreases markedly from 1.51 for the pristine PR/QF composite to 0.957 for the 7.5% POSS-PR/QF sample, indicating that POSS incorporation significantly promotes graphitic ordering within the carbonaceous char.

This enhancement can be attributed to the catalytic and templating effects of silicon-containing species released from POSS during pyrolysis. These species facilitate atomic rearrangement and crosslink reorganization within the carbon matrix, promoting the development of compact, ordered, and thermally stable graphitic domains [41,55]. Consequently, the improved structural order of the char enhances the thermal insulation and mechanical integrity of the ablation layer. This result highlights the crucial role of POSS in regulating microstructural evolution and improving the ablation performance of phenolic-based composites.

## 4. Conclusions

In this study, a novel low-density phenolic aerogel/quartz fiber composite modified with POSS was successfully developed through a thiol–ene click reaction coupled with a sol–gel process. The covalent integration of POSS into the phenolic matrix effectively prevented nanoparticle aggregation and introduced a dual-curing mechanism, resulting in a highly crosslinked organic–inorganic hybrid network. The 7.5%POSS-PR/QF composite exhibited a 114% increase in tensile strength, along with significantly improved thermal stability and oxidation resistance. Meanwhile, its thermal conductivity (0.0619 W/(m·K)) and ablation rates were markedly reduced, indicating excellent thermal insulation and ablation performance.

Mechanistic analysis revealed that POSS reinforces the polymer network, lowers the curing activation energy, and undergoes in situ ceramification at elevated temperatures to form SiO_2_/SiC protective layers. These ceramic phases, together with the catalyzed graphitization of the carbonaceous char, generate a compact and thermally stable barrier that effectively resists heat and oxidative attack. This work provides valuable molecular-level insights. Based on a comparison with the performance of similar materials reported in the literature (Table 3), it is evident that the composites developed in this work achieve an excellent balance of properties while maintaining a low density. Specifically, the composites exhibit high mechanical strength, outstanding thermal insulation performance, and adequate ablation resistance. These results demonstrate that the proposed material system provides a promising strategy for the design of next-generation lightweight thermal protection materials that integrate mechanical robustness, thermal insulation, and ablation resistance.

## Figures and Tables

**Table 1:** Curing reaction kinetic parameters of P1 and P2 peaks.

Peak	Kissinger *E*_a_ (kJ/mol)	Ozawa *E*_a_ (kJ/mol)	Average *E*_a_ (kJ/mol)	*n*
P1	73.42	76.39	74.91	0.932
P2	80.42	84.08	82.25	0.930

Kissinger *E*_a_: *E*_a_ calculated from the Kissinger equation; Ozawa *E*_a_: *E*_a_ calculated from the Ozawa equation; *n*: curing exothermic reaction order. Details are showed in the Supplementary Materials.

**Table 2:** Thermal parameters of PR and POSS-PR under nitrogen.

Sample	T_5%_ (°C)	T_10%_ (°C)	T_dmax_ (°C)	R_800_ (%)	R_1000_ (%)
PR	346	421	531	60.3	58.5
2.5%POSS-PR	350	430	542	62.0	61.2
5%POSS-PR	374	470	553	66.8	64.4
7.5%POSS-PR	421	519	555	71.6	67.6
10%POSS-PR	403	500	548	69.7	66.3

T_5%_: Temperature at which mass loss reaches 5%; T_10%_: Temperature at which mass loss reaches 10%; R_800_: Residual mass at 800 °C; R_1000_: Residual mass at 1000 °C.

**Table 3:** Comparison of the materials prepared in this work with similar material systems.

Material	Density (g/cm^3^)	Mechanical Properties	Thermal Conductivity (W/(m·K))	Ablation Rate	Microstructure
This work	0.7	Tensile strength: 63.26 MPa; Bending strength: 61.76 MPa; Compressive strength: 25.48 MPa	0.0619	0.0584 g/s and 0.197 mm/s	Specific surface area: 84.9 m^2^/g; Average pore diameter: 18.1 nm
2.5D quartz fabric reinforced nanoporous phenolic composites [56]	1.32	Tensile strength: 182.0 MPa	0.21	0.041 g/s and 0.120 mm/s	Average pore diameter: 35 nm
Polysilazane-modified phenolic resin aerogel/carbon fiber [57] composites	0.86	Tensile strength: 59.22 MPa; Bending strength: 23.35 MPa; Compressive strength: 8.89 MPa	0.126	0.112 mm/s	Specific surface area: 37.2 m^2^/g; Average pore diameter: 23.5 nm
Phenolic aerogel/quartz fiber composites [58]	0.35	Compressive strength: 4.4 MPa	0.061~0.064	0.075 g/s and 0.5 mm/s	Specific surface area: 55.5 m^2^/g; Average pore diameter: 194.4 nm
Phenolic resin/silicone hybrid aerogel composites [59]	0.356	Compressive strength: 4.08 MPa	0.098	0.073 mm/s	Specific surface area: 58.42~223.95 m^2^/g; Average pore diameter: 20~60 nm

## Data Availability

The original contributions presented in this study are included in the article. Further inquiries can be directed to the corresponding author.

## Supplementary Materials

The following supporting information can be downloaded at: https://www.mdpi.com/article/10.3390/polym18030387/s1: Figure S1. Curing process; Figure S2. GPC column calibration curve; Figure S3. GPC chromatogram; Figure S4. Molecular weight distribution curve; Figure S5. BET adsorption-desorption curves; Figure S6. Changes in specific surface area of phenolic resins with different POSS; Figure S7. Porosity changes of phenolic resins with different POSS contents; Figure S8. Changes in average pore size of phenolic resins with different POSS; Table S1. Molecular weight statistical results.
