# Supplementary material for: Preparation of Phenolic Aerogel/Quartz Fiber Composites Modified with POSS: Low Density, High Strength and Thermal Insulation
Source: Polymers (Basel). 2026 Jan 31;18(3):387. doi: 10.3390/polym18030387 (PMC12899982; doi:10.3390/polym18030387)
Supplement: Supplementary file 1 [file polymers-18-00387-s001.zip › polymers-4068842-supplementary.pdf]

Supplementary Materials for

Preparation of POSS-modified phenolic aerogel/quartz fiber composites: low density, high strength, great thermal insulation and ablation resistance

Authors: Xiang Zhao[1], Dayong Li[1]*, Meng Shao[1], Guang Yu[1], Wenjie Yuan[1], Junling Liu[1], Xin Ren[1], Jianshun Feng[2], Qiubing Yu[1], Zhenyu Liu[1], Guoqiang Kong[1], Xiuchen Fan[3]

Author Affiliations:

[1]Shandong Institute of Nonmetallic Materials, Jinan 250031, China

[2]Key Laboratory for Liquid Solid Structural Evolution and Processing of Materials, School of Materials Science and Engineering, Shandong University, Jinan 250061, China

[3]Beijing Xinfeng Aerospace Equipment Co., Ltd., Beijing 100089, China

Corresponding Author: Dayong Li

Address: Shandong Institute of Nonmetallic Materials, Jinan 250031, China

Email: 15965639188@163.com

S1 Equation parameter fitting

Based on the Kissinger (1), Ozawa (2), and Crane (3) equations, the apparent activation energy (E_a) and reaction series of the curing reactions were calculated as follows:

$$\ln\left(\frac{\beta}{T_p^2}\right) = \ln\left(\frac{AR}{E_a}\right) - \frac{E_a}{R} \cdot \frac{1}{T_p} \tag{1}$$

$$\ln\beta = \ln\frac{AE_a}{R} - \ln g(a) - 5.331 - 1.052\,\frac{E_a}{RT_p} \tag{2}$$

$$\frac{d(\ln\beta)}{d(\frac{1}{T_p})} = -\frac{E_a}{nR} - 2T_p \tag{3}$$

Where β is the heating rate (°C/min); T_p is the peak temperature of the curing reaction. E_a is the apparent activation energy of the curing, R is the gas constant, and A is the pre-exponential factor. Based on the DSC results, a reasonable curing procedure was designed as follows: 120 °C/2 h → 145 °C/3 h → 175 °C/2 h → 210 °C/3 h → 230 °C/1 h (Figure. S1).

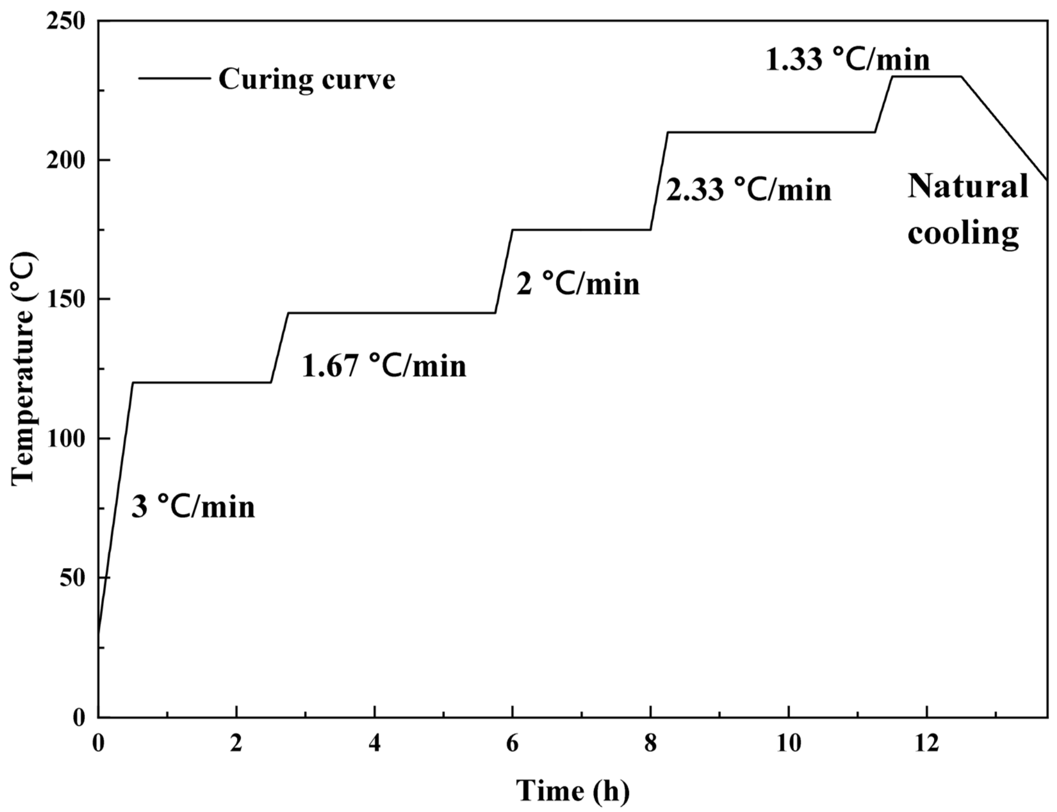

Figure. S1 Curing process

S2 SH-POSS GPC results

Molecular weight-retention time fitting equation after GPC column calibration:

$$\log M = -0.915672t + 11.7027 \quad (6)$$

Where M is the molecular weight and t is the retention time.

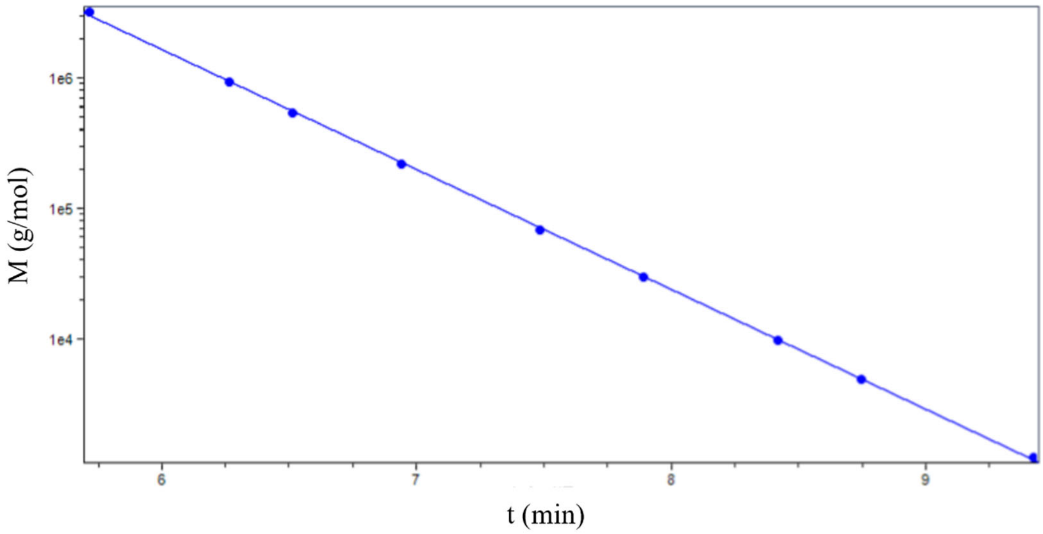

Figure. S2 GPC column calibration curve

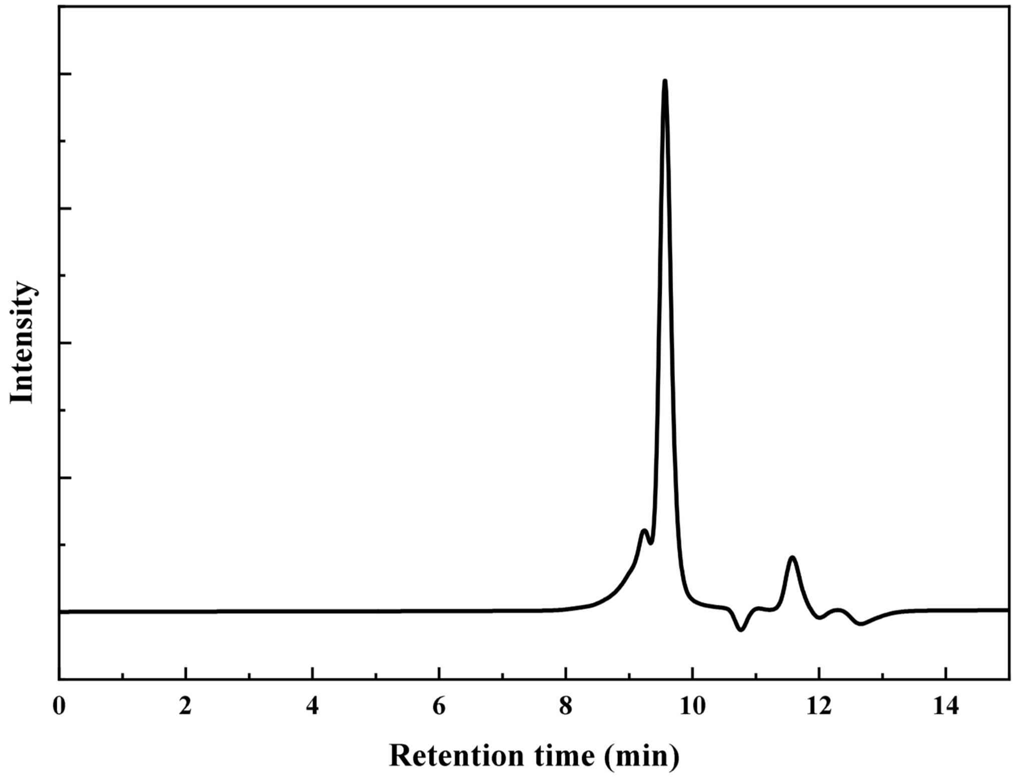

Figure. S3 GPC chromatogram

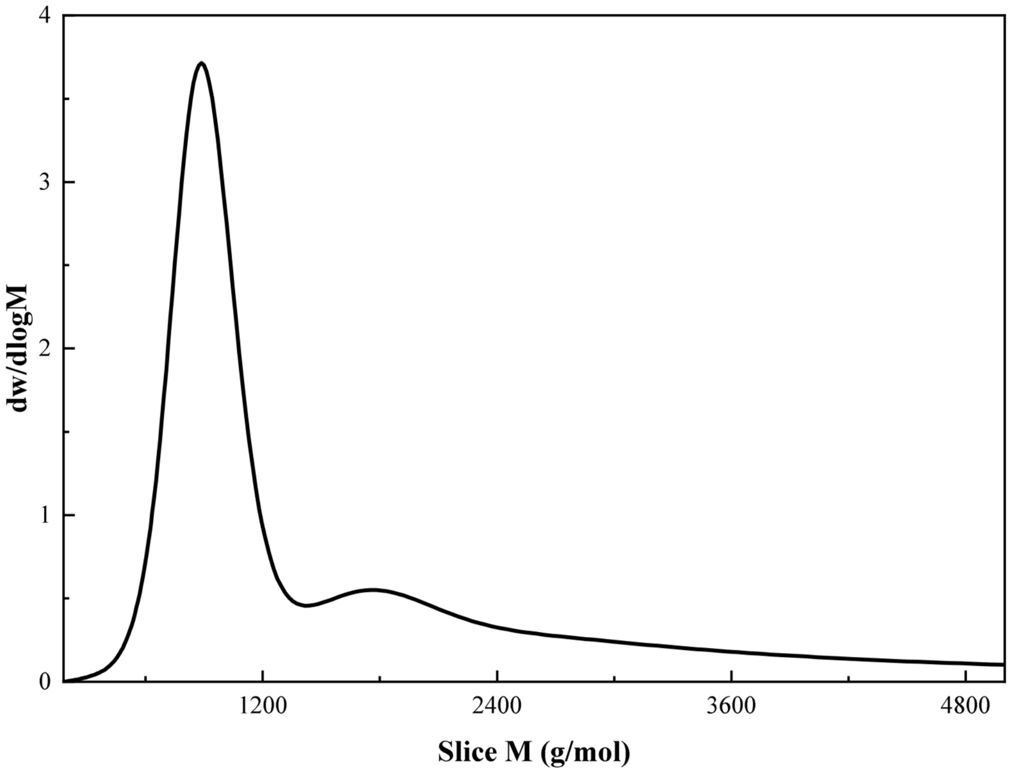

Figure. S4 Molecular weight distribution curve

Table S1 Molecular weight statistical results

Mp (g/mol)	M_n (g/mol)	M_w (g/mol)	M_z (g/mol)	M_{z+1} (g/mol)	M_η (g/mol)	Polydispersity
886	940	1183	2589	6398	1192	1.259

According to the above GPC test results (Figure. S2, S3, S4 and Table S1), the actual weight-average molecular weight of SH-POSS (1183) is close to the theoretical value (1016), which proves the successful synthesis of SH-POSS; combined with the polydispersity coefficient (1.259) and the molecular weight distribution curve, it shows that the product contains a small amount of non-T8-POSS, such as T7-POSS, T10-POSS, etc.

S3 PR and POSS-PR BET analysis

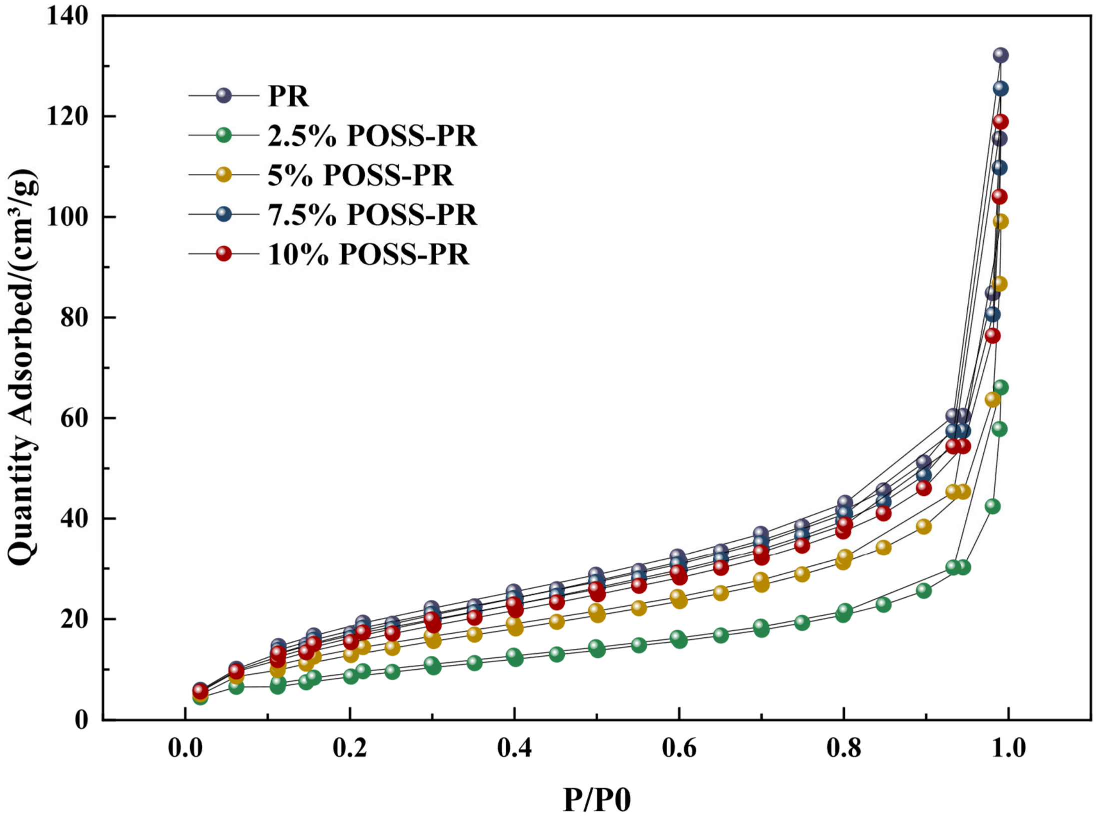

Figure. S5 BET adsorption-desorption curves

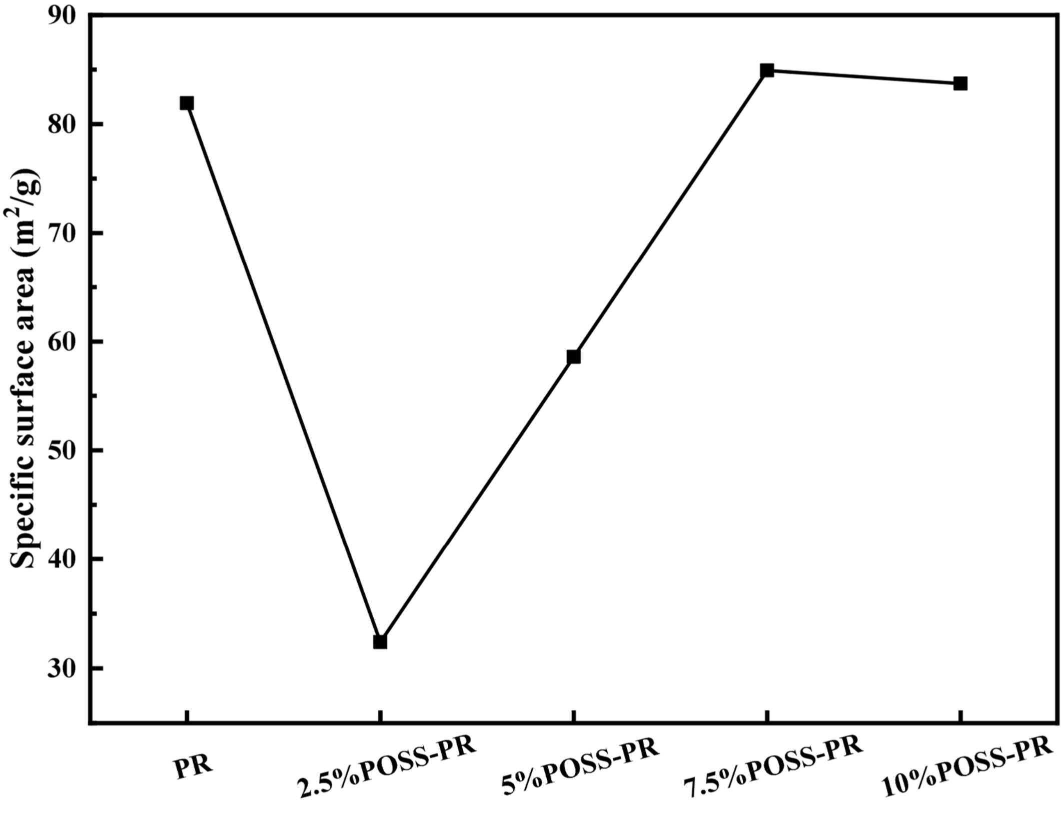

Figure. S6 Changes in specific surface area of phenolic resins with different POSS contents

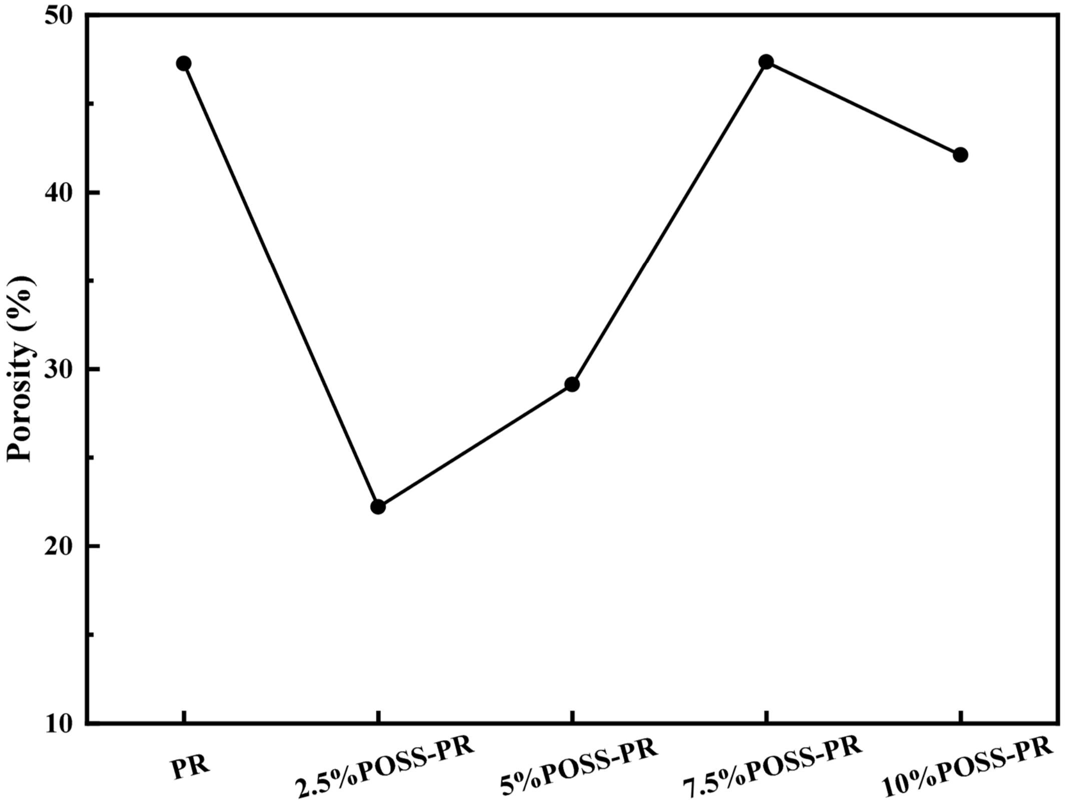

Figure. S7 Porosity changes of phenolic resins with different POSS contents

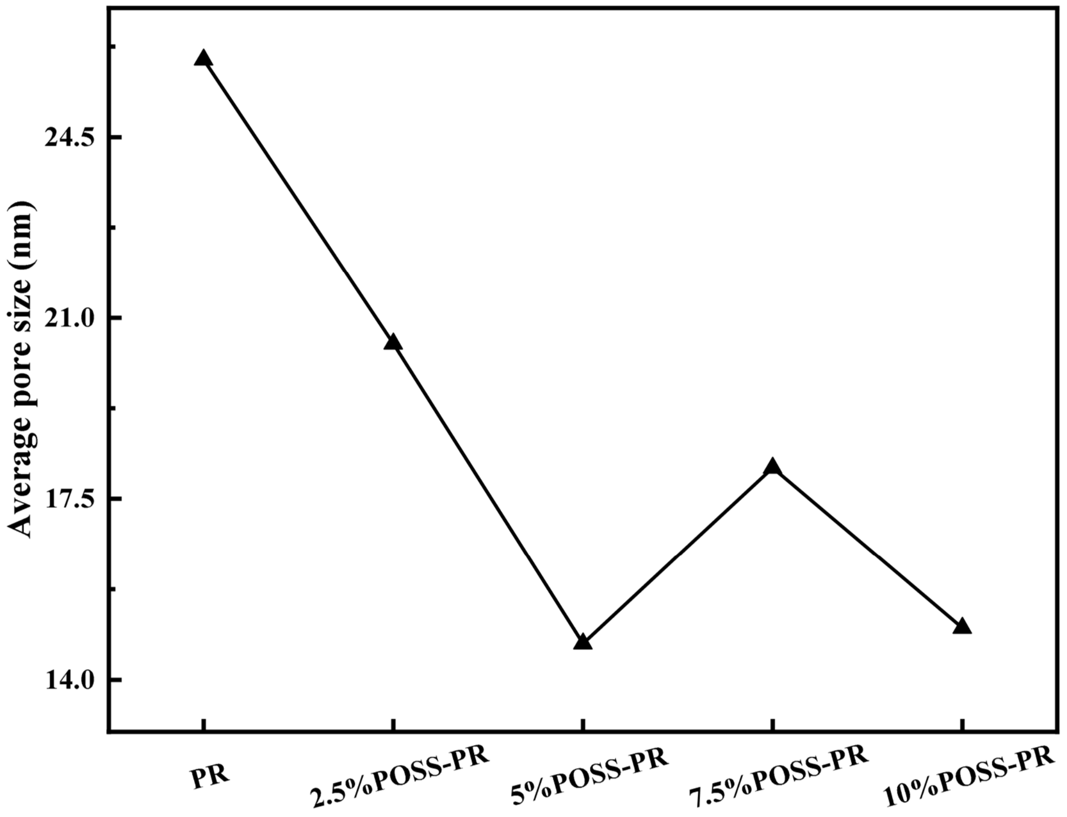

Figure. S8 Changes in average pore size of phenolic resins with different POSS contents

The N_2 adsorption-desorption curves of PR and POSS-PR are shown in Figure. S5. As the POSS content increases, the average pore size gradually decreases (Figure. S8). This is due to the POSS molecules occupying the voids within the phenolic porous network. However, the porosity and specific surface area show a pattern of first decreasing and then increasing (Figure. S6 and S7). This is because as the POSS content increases, the rigid structure of the POSS molecules exerts its support and expansion effect on the phenolic porous network, causing the hybrid network to expand further. The porosity-increasing effect caused by this expansion outweighs the porosity-reducing effect caused by the POSS molecules occupying the pores. Taken together, the higher porosity, specific surface area, and smaller pore size confirm the formation of the phenolic aerogel structure and indicate that the introduction of POSS molecules helps reduce the pore size of the phenolic aerogel material and increase the porosity and specific surface area.
